# P04.42. Use of complementary and alternative medicine among adults with neuro-psychiatric symptoms common to mild traumatic brain injury

**DOI:** 10.1186/1472-6882-12-S1-P312

**Published:** 2012-06-12

**Authors:** M Purohit, R Wells, S Bertisch, R Zafonte, R Davis, R Phillips

**Affiliations:** 1 Beth Israel Deaconess Medical Center/Harvard Medical School, Boston, USA; 2 Brigham and Women's Hospital/Faulkner Hospital/Harvard Medical School, Boston, USA; 3 Spaulding Rehabilitation Hospital/Harvard Medical School, Boston, USA

## Purpose

One in three adults uses complementary and alternative medicine (CAM) annually in the United States. However, the pattern of CAM use among adults with neuropsychiatric symptoms commonly reported by patients with mild traumatic brain injury (mTBI), a serious public health concern, is not well studied.

## Methods

We analyzed data from the 2007 National Health Interview Survey (n=23,393) to compare CAM use between adults with and without neuropsychiatric symptoms common to mTBI. Symptoms included self-reported anxiety, depression, insomnia, headaches, memory deficits, attentional deficits, and excessive sleepiness. CAM use was defined as use of mind-body (e.g., meditation), biological (e.g., herbs), manipulation (e.g., massage) therapies, and alternative medical systems (e.g., Ayurveda), within the past 12 months. We estimated prevalence and reasons for CAM use in patients with and without neuropsychiatric symptoms. We also explored variations in CAM use by the number of symptoms. Multivariable logistic regression was performed to examine the association between neuropsychiatric symptoms and CAM use after adjustment for sociodemographic characteristics, illness burden (e.g,. fibromyalgia, low back pain), access to care, and health habits.

## Results

Adults with neuropsychiatric symptoms had higher CAM use compared to adults without neuropsychiatric symptoms (44% vs. 30%, p<0.001); prevalence increased with increasing number of symptoms (p-value for trend <0.001, table below). Differences persisted after adjustment (table below). Twenty percent used CAM because standard treatments were either too expensive or ineffective; 25% used CAM because it was recommended by a provider.

**Table 1:** 

Number of Symptoms	aOR	95% CI	
0	1.00		
1	1.43	1.32	1.56
2	1.70	1.51	1.91
≥3	1.77	1.56	2.01

## Conclusion

More than 40% of adults with neuropsychiatric symptoms observed in mTBI used CAM. An increasing number of symptoms was associated with increased use. Future research is needed to understand the use, efficacy, and safety of CAM in mTBI patients.

